# Transplantation of bone marrow derived macrophages reduces markers of neuropathology in an APP/PS1 mouse model

**DOI:** 10.1186/s40035-019-0173-9

**Published:** 2019-10-14

**Authors:** Luís Costa-Marques, Katrin Arnold, Marie-Christine Pardon, Christiane Leovsky, Samantha Swarbrick, Claire Fabian, Alexandra Stolzing

**Affiliations:** 1 Centre for Biological Engineering, School of Mechanical, Electrical and Manufacturing Engineering, Loughborough University, Epinal Way, Loughborough, UK; 2 Fraunhofer Institute for Cell Therapy and Immunology, Leipzig, Germany; 3 School of Life Sciences, Queens Medical Centre, University of Nottingham, Nottingham, UK; 4 Leipzig University, Leipzig, Germany

**Keywords:** Amyloid beta, Microglia, Alzheimer’s disease, Cell therapy, Neuroinflammation

## Abstract

**Background:**

We investigated early hallmarks of putative therapeutic effects following systemic transplantation of bone marrow derived macrophages (BM-M) in APP/PS1 transgenic mice.

**Method:**

BM-M were transplanted into the tail vein and the animals analysed 1 month later.

**Results:**

BM-M transplantation promoted the reduction of the amyloid beta [37**-**42] plaque number and size in the cortex and hippocampus of the treated mice, but no change in the more heavily modified pyroglutamate amyloid beta E3 plaques. The number of phenotypically ‘small’ microglia increased in the hippocampus. Astrocyte size decreased overall, indicating a reduction of activated astrocytes. Gene expression of interleukin 6 and 10, interferon-gamma, and prostaglandin E receptor 2 was significantly lower in the hippocampus, while interleukin 10 expression was elevated in the cortex of the treated mice.

**Conclusions:**

BM-M systemically transplanted, promote a decrease in neuroinflammation and a limited reversion of amyloid pathology. This exploratory study may support the potential of BM-M or microglia-like cell therapy and further illuminates the mechanisms of action associated with such transplants.

## Background

Microglia are a key protagonist in the central nervous system (CNS) immune system. They regulate amyloid beta (Aβ) content through phagocytosis, playing a central role in the pathology and progression of Alzheimer’s Disease (AD) [1–3].

Depending on the surrounding stimuli, microglia may assume either a more pro-inflammatory (M1) or anti-inflammatory (M2) state [4], though this might be a fluid continuum [5]. Short-term microglial activation is a natural part of neuroprotection in the brain, contributing to Aβ clearance [6], whereas chronic activation has been associated with promoting neurodegenerative disorders such as AD [7–9]. As AD progresses microglia chronic activation becomes detrimental and triggers a progressive damaging cycle to the brain [9].

Therefore, an adjustment of dysfunctional microglia cells via, for example, the replenishment with young functional microglia, can be an effective therapeutic strategy. A few early reports have explored this idea [10–13], although with different cell types, delivery methods and AD animal models.

The first study was in rats using stereotactic amyloid injections and using brain-derived microglia [10]. Stereotactic inject of cells does induce damage in the brain and leads to inflammation un-related to the disease. The second study used monocytes which are less differentiated compared to our BM-M and known to be phagocytic less active [11]. The third study is close to the aim of our study as bone marrow derived microglia were transplanted, however this study explored the priming with IL-4 to derive M2-like microglia. The cells were not characterized beyond the use of CD206 as an M2 marker and no cell tracking data was provided [12]. In addition, this was again using a rat model in which stereotactic injections were used. Changes observed in this model does not capture the element of aging, focusing solely on the role of amyloid alone as a model for AD.

The aim of this study was on using the most appropriate AD model, cell production that is easily scalable and translatable as well as using well characterized cells.

## Material and methods

### Animals

Transgenic mice used were overexpressing human amyloid precursor protein (APPKM670/671NL) and presenilin-1 (PS1L166P) under Thy-1 promoter control (age 16-19 months) (source: Dr. Mathias Jucker, Hertie-Institute for Clinical Brain Research, University of Tübingen, Germany). Mice were housed according to local regulations. Injections were performed by Dr. Pardon (Animal License Holder) under the project number 40/3601. ‘Donor’ C57Bl/6 male young mice (3 months) for obtaining microglia were bred in the Leipzig University animal facilities (Landesdirektion Sachsen, License Number T 19/14).

### BM-M differentiation from bone marrow

Femur and tibiae of young C57BL/6 mice were isolated with a scalpel. Each bone was placed in an Eppendorf tube and centrifuged, 400 g for 1 min at 4 °C. The isolated pellet of bone marrow of one femur and one tibia of the same mouse was pooled and resuspended with DMEM low glucose (Gibco) with 1% penicillum/streptomycin (Gibco) and 10% FBS (Hyclone). Afterwards, the cells were cultured in 100 mm petri dishes at 37 °C, 5%CO_2_ and 20%O_2_ with medium change after 3 days. After 10 days the non-adherent bone marrow-derived stem cells were used for differentiation into microglia-like cells. In detail, the cell suspension was centrifuged, 200 g for 5 min at room temperature. The pellet was resuspended with a medium mix and placed on a 100 mm petri dish. This medium mix included one-part DMEM (low glucose + 10%FBS) with 20 ng/ml GM-CSF (Peprotec) and one part astrocyte-conditioned DMEM medium, obtained from culturing astrocytes over 24 h in this medium. The differentiation took an additional 7 days without medium change by incubation at 37 °C, 5%CO_2_ and 20%O_2_. All adherent cells were detached using trypsin and used for transplantation.

### Flow cytometry of microglia

Briefly, cells were trypsinized and filtered through a 40 μm filter and fixed with 2% (v/v) paraformaldehyde (Pierce, 16% Formaldehyde, Methanol-free) for 15 min at room temperature. Cells were centrifuged at 500 g for 5 min and washed with phosphate buffered saline (PBS). Subsequently a blocking step with 0.5% (v/v) BSA for 30 min at room temperature. Cells for CD68 staining were permeabilized with 0.2% (v/v) Tween20 before blocking. Cells were incubated for 30 min at room temperature with fluorescence-labeled antibodies CD45-PE (1:100; Myltenyi-REA737, 130-110-659), CD11b-PE (1:100; Myltenyi-REA592, 130-109-285); F4/80-PE (1:100; Myltenyi-REA126, 130-102-422), CD68-PE (1:300; Myltenyi-FA11, 130-102-614), CD80-PE (1:300; Myltenyi-16-10A1, 130-102-613), CD86 (1:300; Myltenyi-PO3.3, 130-102-604); MHCII (1:300; Myltenyi- M5/114.15.2, 130-102-186), CD206-PE (1:300; Life technology- MR6F3, 12-2061-82), CD16/32-PE (1:100; Myltenyi-REA377, 130-107-039), CD64-PE (1:100; Myltenyi-REA286, 130-103-808), CD169-PE (1:100; Myltenyi-REA197, 130-104-953), CD204-PE (1:100; Myltenyi-REA148, 130-102-328), Dectin-PE (1:100; Myltenyi-REA154, 130-102-284), CD124-PE (1:100; Myltenyi-REA235, 130-102-710), rat IgG2b isotype-PE (1:300; Myltenyi, 130-102-663), hamster IgG2 isotype (1:300; Thermo Fisher, 12-4888-81) and REA isotype-PE (1:100; Myltenyi-130-104-628). Cells were washed again and fluorescence was measured using the BD Influx. Dot blot graphs were created using BD FACS™ Software. A gate for total cells was set on the FSC vs. SSC plot. PE was analysed using a 561 nm laser and a BP 585/29 filter. Marker expression analysis of the gated cells was done using the appropriate isotype as negative control.

### Transplantation

1 × 10^6^ BM-M cells in 100 μl PBS were injected into the tail vein of AD mice (16-19 months, *n* = 12). The control AD mice (*n* = 12) were injected with 100 μL of PBS.

### Tissue preparation

After 28 days from transplantation the mice were sacrificed and the brains were isolated for histology (BM-M, *n* = 6; control *n* = 6) and biochemical analysis (BM-M, *n* = 6; control *n* = 6). For biochemistry, mice were perfused transcardially post mortem with 0.9% (v/v) NaCl. Brains were removed, divided into regions (hippocampus, cortex, brainstem) and stored in peqGOLDTriFast™ (PeqLab, 30-2040, Erlangen, Germany) at − 80 °C until further use. For histology, mice were perfused transcardially post mortem with 0.9% (w/v) NaCl followed by fixative containing 4% (v/v) paraformaldehyde and 0.1% (v/v) glutaraldehyde in 0.1 M phosphate buffer (pH 7.4). Brains were removed and immersion-fixed overnight in the same fixative at 4 °C. Brains were cryoprotected in 30% sucrose in 0.1 M phosphate buffer (pH 7.4) with 0.1% (w/v) sodium azide and cryosectioned into 20 μm coronal sections with a cryomicrotome (Shandon CryotomeSME, Thermo Scientific) in frontal plane. Sections were collected in 0.1 M phosphate buffer (pH 7.4) with 0.1% sodium azide and stored at 4 °C until analysis.

### Immunohistochemistry

Brain slices were washed twice with PBS-0.05% (v/v) Tween20 and incubated with blocking solution - 2% bovine serum albumin (Serva, #47330), 0.3% powdered milk (Applichem, #A0830) and 0.5% donkey serum (Jackson ImmunoResearch, #017-000-001) for 30 min at room temperature. Slices were incubated with primary antibodies diluted in blocking buffer: anti-Iba-1 rabbit (1:200, Wako), β-amyloid (1:100, XP® rabbit mAb, 8243S New England Biolabs), Pyro-GluAbeta pE3 (1:400, biotinylated monoclonal, Synaptic systems, 218,011 BT), Glial Fibrillary Acidic Protein (1:500, GFAP, Polyclonal, DAKO Z0334292) overnight at 4 °C in the dark. Slices were washed with PBS followed by incubation with the secondary antibody NorthernLights™ Anti-rabbit IgG-NL637 (1:200, NL005), anti-mouse IgG NL557 (1:200, NL007) and anti-streptavidin (1:5000, NL999) for 1 h at RT. Slices were then incubated for 30 min with DAPI (1:10,000; Sigma) at RT. Stained brain slices were mounted with ProLong® Gold Antifade Mountant (Molecular Probes, P36934).

### Microscopy and image processing

Images from the immunostained mice brain slides were captured with a Carl Zeiss AxioScan.Z1 slide scanner microscope, using ZEN blue (2012) software and acquired under the same exposure time settings for all the brains. Scan was performed under the 20x objective, at the wavelengths 545 nm (Zeiss 43 HE filter), 620 nm (Chroma ET49006 filter) or 365 nm (Zeiss 49 HE filter) depending on the fluorophore. From each mouse, 3 brain sections were scanned and analysis was performed in the anterior, middle and posterior cortex, hippocampus and brainstem. ZEN blue (2012) slide scan software was used to visualize the sections images and to minimally adjust the image background. The number and area of Aβ positive plaques, Iba-1 positive and GFAP positive cells were further evaluated and quantified using ImageJ software (tool of the public domain, https://imagej.nih.gov/ij/). Images were initially converted to 8-bit channels, scale was set from pixels to μm, threshold was established independently for each mouse and the region of interest was manually outlined and measured. The analyze particles function on Image J (Aβ plaques size set: 100-infinity μm^2^; microglia size set: 100-infinity μm^2^; circularity 0.00-1.00) was used to determine number and area of plaques and microglia cells. Plaque number per mm^2^ was calculated using the counted number of plaques divided by the total measured region. Plaque size was calculated by dividing the total area covered of plaques by plaque number [14]. The same number and size calculations were made for subgroups of small, medium and large plaques (100-500 μm^2^, 500-1500 μm^2^ and > 1500 μm^2^, respectively) individually for total cortical, hippocampal or brainstem region. Calculations were performed in an unbiased manner by an investigator blind to the treatment conditions of the samples.

### RNA isolation and qRT-PCR

RNA was extracted from hippocampus, cortex and brainstem using peqGOLDTriFastTM (PeqLab, 30-2040, Erlangen, Germany) reagent according to the manufacturer’s instructions. RNA was treated with DNaseI (Life Technologies, EN0521) to remove genomic DNA contamination. This procedure was followed by cDNA synthesis using Superscript III-reverse transcriptase (Life Technologies, 18,080,085) and Oligo (dT)_18_-Primers (Thermo Scientific, SO132) at 50 °C for 1 h. cDNA was used as PCR template in an 1:10 dilution and each sample run in triplicate. Quantitative PCR was performed on StepOnePlus™ Real-Time PCR System (Applied Biosystems) using Express SYBR GreenER qPCR Supermix Universal (Life technologies, 1,178,401 K), 0.2 μM primer each on the DNA (primers as published by us before) with the following cycle conditions: primary denaturation at 95 °C for 3 min at 95 °C, 35 cycles with 30s at 95 °C, 30s at 60 °C and 30s at 72 °C followed by fluorescence measurement. Absolute quantification was performed for every single gene with three technical repeats per sample. Serial dilutions of plasmid controls with known molecule concentrations were used as a positive control and to generate standard curves. Expression of target genes were normalized using 36B4 (large ribosomal protein P0, RPLP0) as reference gene.

### Elisa

Glial fibrillary acidic protein (GFAP) (NS830, Merck), mouse triggering receptor expressed on myeloid cells 2 (TREM-2) (CSB-EL024405MO, Generon) and acetylcholinesterase (AChE) (E-EL-M2637, Generon) were quantified by enzyme-linked immunosorbent assay (ELISA) kit, as per the manufacturer’s instructions. Protein isolation was done by ethanol-bromochloropropane-water method according to Chey et al. [15] followed by further detergent removal with Compat-Able™ BCA Protein Assay Kit (cat no. 23229, Thermofisher Scientific). The colorimetric signal was quantified using a plate reader (Fluostar Omega, BMG Labtech). Protein concentrations were calculated from the absorbance data (at 450 nm) and were normalized to total protein content determined by Pierce™ BCA Protein Assay Kit (cat no. 23225, Thermofisher Scientific).

### Statistical analysis

Statistical analysis was performed using the GraphPad Prism 6 (GraphPad Software). Data were tested for normality using the D’Agostino-Pearson omnibus normality test. Values between two groups were compared by the two-tailed unpaired Student’s t test for normally distributed data. *P* values of *p* < 0.05 (*), *p* < 0.01 (*) and *p* < 0.01 (***) were considered significant.

## Results

### BM-M characterization

**Fig. 1:**
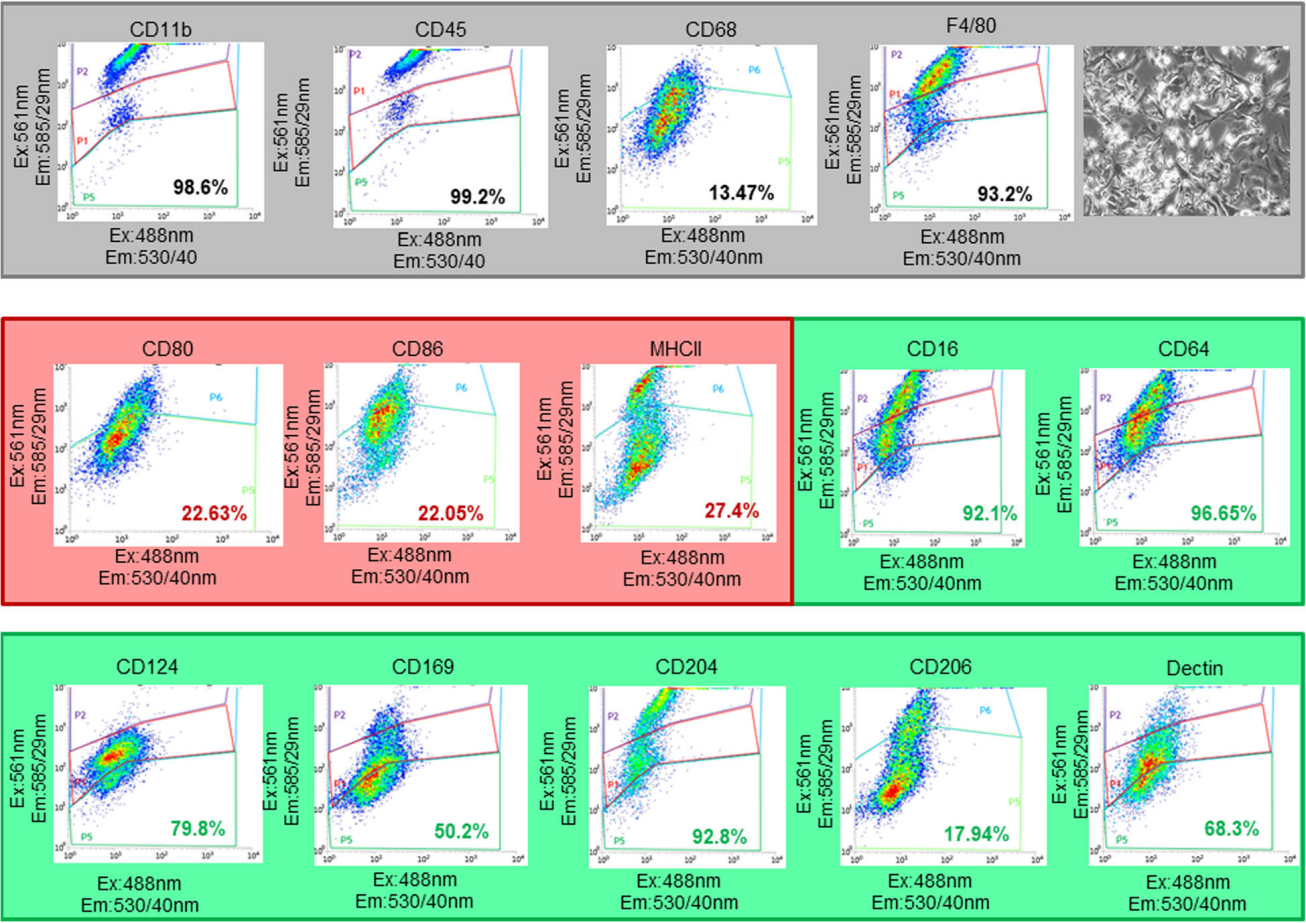
Characterization of BM-M phenotype by flow cytometry. BM-M were positive for CD11b, CD45, CD68, CD206 and F4/80, which are general microglia markers. Levels of M2 specific microglia markers (CD16, CD64, CD169, CD124, CD204 and dectin) were higher than M1 markers (CD80, CD86, and MHCII) indicating the prevalence of a microglia-M2 phenotype. At the top right a representative image of the transplanted BM-M is shown

Prior to transplantation, the BM-M were exposed astrocyte conditioned medium and cell viability measurement was performed (BM-M viability> 90%). These conditioned BM-M were positive for CD11b, CD45, CD68 and F4/80, which are general microglia markers (Fig. 1). In addition, we stained the cells for M1 and M2 markers and found the BM-M to be mainly of a microglia-M2 phenotype (CD16, CD64, CD169, CD124, CD204, CD206 and dectin). M1 markers (CD 80, CD86, and MHCII) expression levels were low (< 30%).

### Aβ_[37-42]_ numbers and size

**Fig. 2:**
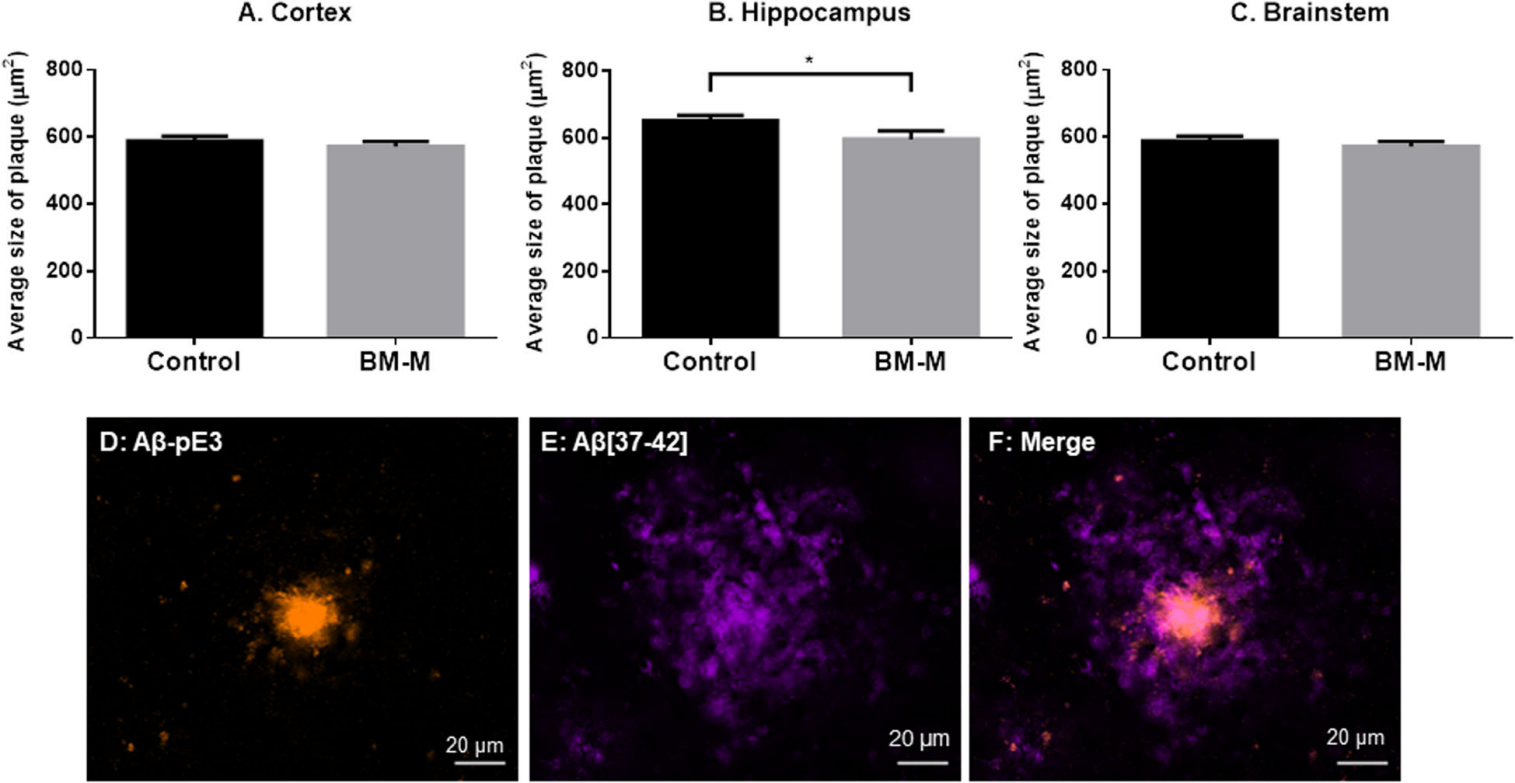
Average size of Aβ[37-42] plaques in cortex, hippocampus and brainstem. BM-M transplantation decreases Aβ plaques size in the hippocampus of the APP/PS1 treated mice (*n* = 6) compared to control group (*n* = 6) (**a-c**). Representative co-staining of Aβ_[37-42]_ (purple) and Aβ-pE3 plaques (orange), showing the dense Aβ-pE3 plaque modification localized in the centre of a Aβ_[37-42]_ plaque (**d-f**). Bar graphs display the mean ± SEM (error bars) of plaque and student’s *t*-test was used for statistical analysis (*p < 0.05)

**Fig. 3:**
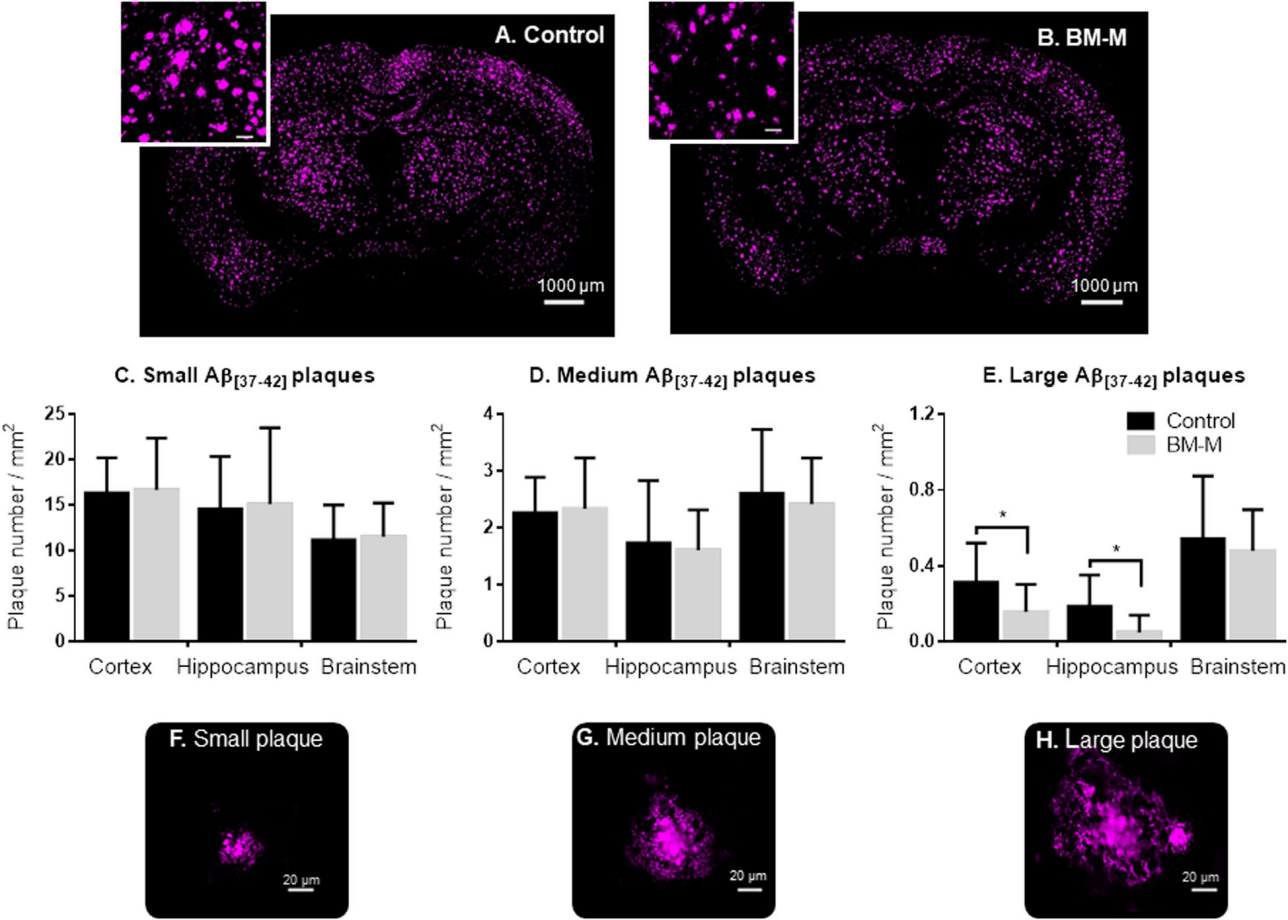
Aβ_[37-42]_ plaques number decrease in cortex and hippocampus of APP/PS1 mice treated with BM-M. **a, b** Representative Aβ_[37-42]_ plaques immunostaining comparison between PBS injected mice (control) and BM-M treated mice, showing less large plaques in transplanted animals. **c-e** Small, medium and large plaque number per mm^2^ in cortex, hippocampus and brainstem comparison between control and BM-M treated mice showing a reduction of larger plaque in cortex and hippocampus. **f-h** Representative images of different plaque sizes stained by immunohistochemistry are shown. Bar graphs display the mean ± SEM (error bars) of plaque (*p < 0.05)

Abeta_[37-42]_ covers the bulk of amyloid in AD brains in this mouse model [16] and was used to quantify the changes after BM-M transplantations. Twenty-eight days after administration of BM-M or PBS, mice brains were evaluated for changes in Aβ deposition. The number and size of plaques were quantified in cortex, hippocampus and brainstem individually as these regions are differently loaded with amyloid plaques in this mouse model [17]. We found that transplantation of BM-M resulted in 9% (*p* < 0.05) reduction of plaque size in the hippocampus only (Fig. 2). Although we could not detect a change in total Aβ_[37-42]_ plaque numbers, our data shows that transplantation resulted in a reduction of the number of larger plaques (> 1500 μm^2^) particularly in the cortex (50%, *p* < 0.03) and hippocampus (70%, *p* < 0.02) (Fig. 3). These results suggest that there is an effect mediated by the transplanted BM-M on the Aβ_[37-42]_ plaques and that this is more pronounced in the hippocampus and for larger plaques.

### Aβ-pE3 numbers and size

**Fig. 4:**
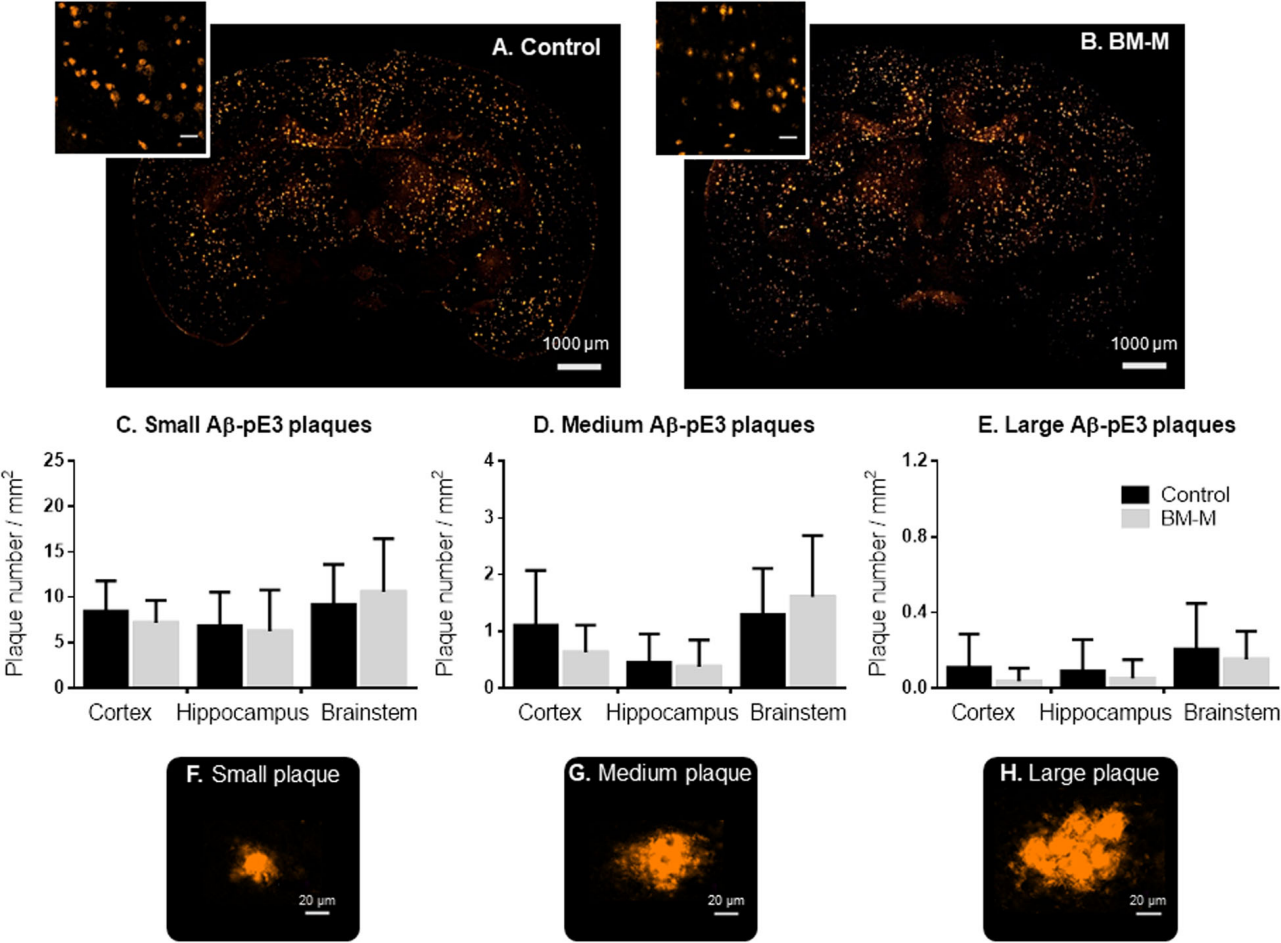
Aβ-pE3 plaques number in cortex and hippocampus of APP/PS1 mice treated with BM-M. **a, b** Representative Aβ-pE3 plaques immunostaining comparison between PBS injected mice (Control) and BM-M treated mice, showing no significant change in Aβ-pE3 plaque subgroup (**c-e**). Small, medium and large plaque number per mm^**2**^ in cortex, hippocampus and brainstem comparison between control and BM-M treated mice (**f-h**) Representative images of different plaque sizes stained by immunohistochemistry. Bar graphs display the mean ± SEM (error bars) of plaque

To evaluate the ability of the transplanted BM-M to invade the core of amyloid plaques we also quantified one of the modified amyloid forms known to be resistant to proteolysis and often found in the center of plaques - the pyroglutamate-modified Aβ peptide (Aβ-pE3) [18]. Double staining of Aβ_[37-42]_ and Aβ-pE3 clearly confirms this composition of amyloid plaque types in our mouse model (Fig. 2f). No differences were found regarding the number or size of Aβ-pE3 plaques, neither in total brain area nor in the different brain regions analyzed (Fig. 4), which indicates that under the current transplantation conditions, BM-M cells do not have an impact on this subgroup of Aβ plaques (Fig. 4f–h).

### Microglia (IBA-1^+^) numbers

We evaluated different microglia cell sizes – small microglia cells (< 300 μm^2^), cluster of microglia I (300-900 μm^2^) and cluster of microglia II (> 900 μm^2^).

**Fig. 5:**
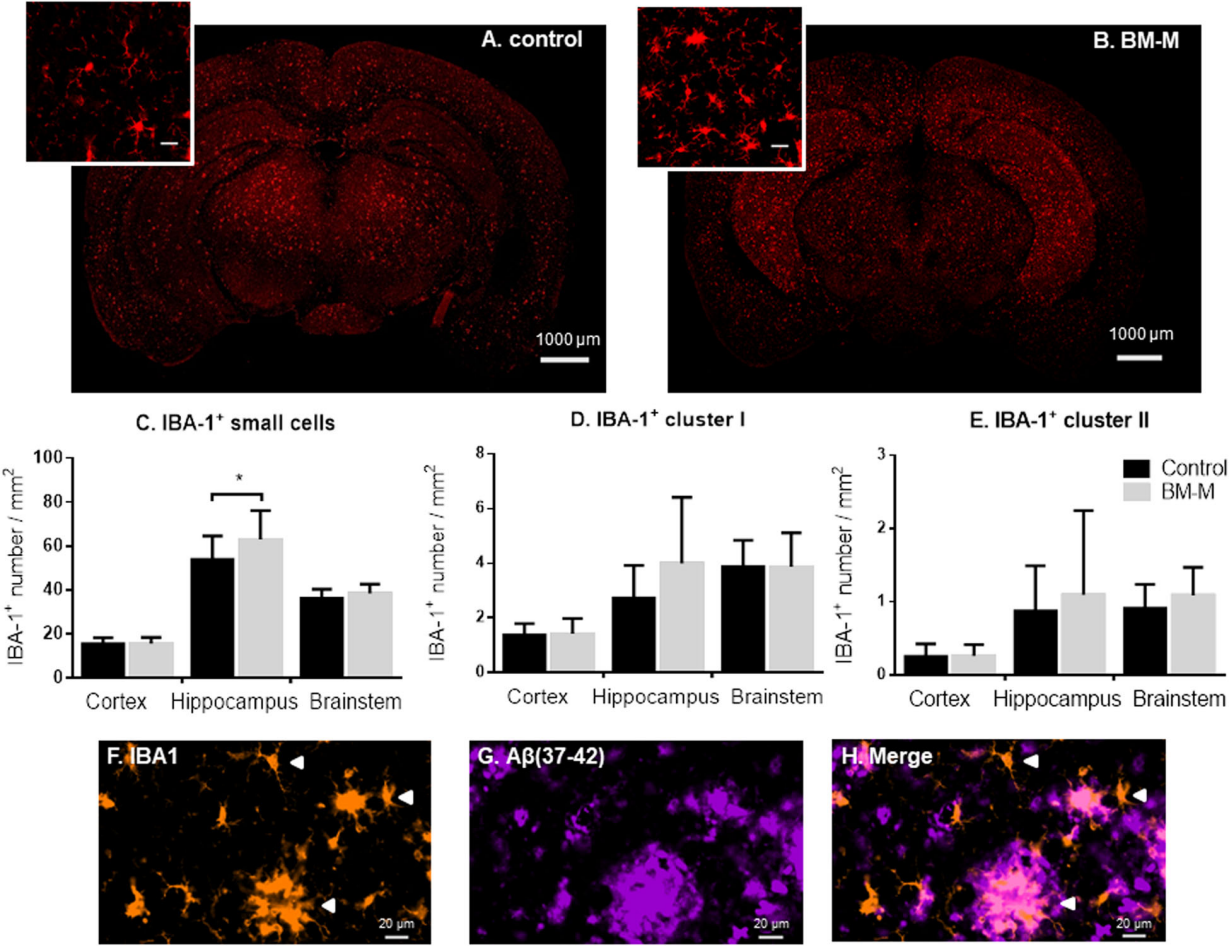
Small IBA-1+ cells number increase in hippocampus of APP/PS1 mice treated with BM-M. **a, b** Representative IBA-1 immunostaining comparison between PBS injected mice (Control) and BM-M treated mice. **c-e** Small IBA-1 microglia cells, medium sized cluster of microglia (cluster I) and large sized clusters of microglia number per mm^**2**^ in cortex, hippocampus and brainstem comparison between control and BM-M treated mice showing an increase of small microglia cells in the hippocampus of treated mice. **f-h** Representative images of different microglia sizes and clusters co-stained with Aβ_[37-42]_ plaques showing clusters of microglia around plaques and small microglia cells not associated Aβ. Bar graphs display the mean ± SEM (error bars) (*p < 0.05)

BM-M transplantation was associated with an increased incidence of small microglia but had no effect on microglia clusters (associated with high microglia activation) (Fig. 5a–e). Our data shows an increase compared to controls in the number of small microglia in the hippocampus only (20%, *p* < 0.03 - Fig. 5c), which links to the results shown above for Aβ plaque content. Observationally, when co-staining IBA-1 with Aβ_[37-42]_, the association of large microglia clusters with large Aβ plaques was evident, while small soma cells were generally not associated with plaque co-localization (Fig. 5f–h).

### Astrocytes

**Fig. 6:**
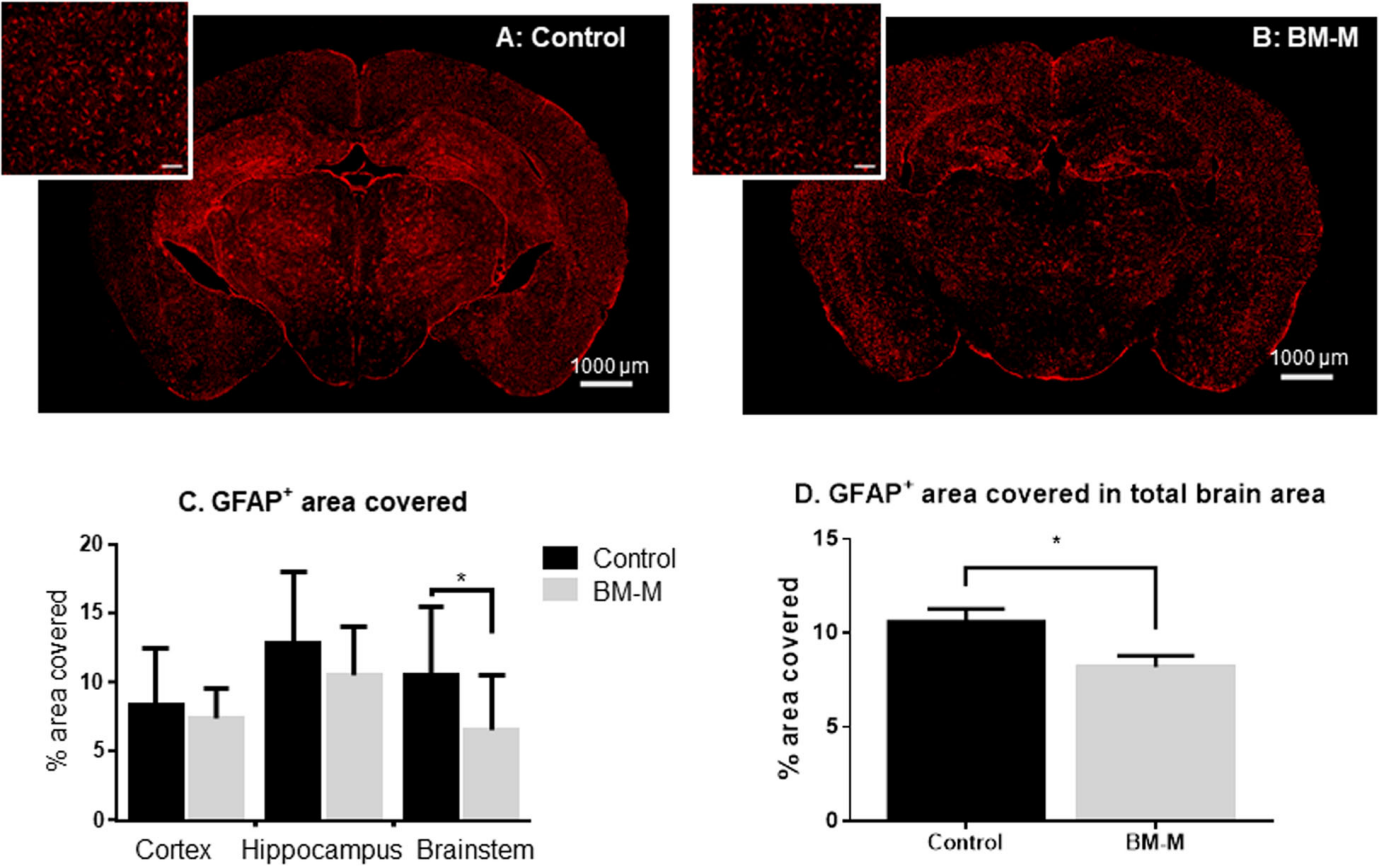
GFAP (astrocytes) area covered decreases on APP/PS1 mice treated with BM-M. **a, b** Representative GFAP immunostaining comparison between PBS injected mice (control) and BM-M treated mice showing less area covered by GFAP in the brains of the transplanted mice group. **c** Percentage of GFAP+ area covered in cortex, hippocampus and brainstem individually, shows a significant decrease in the brain stem area of BM-M treated mice. Bar graphs display the mean ± SEM (error bars) (*p < 0.05). **d** Percentage of GFAP+ area covered in total brain area shows a significant reduction upon BM-M transplantation

Using immunohistochemistry, the total area covered by astrocytes showed a decrease in the treated animals compared to controls (23%, *p* < 0.02) particularly in the brainstem (37%; *p* < 0.03) (Fig. 6). The decrease indicates a reduction of astrocyte activation which in turn is associated with reduced neuroinflammation [19] .

### BM-M effect on gene and protein expression

**Fig. 7:**
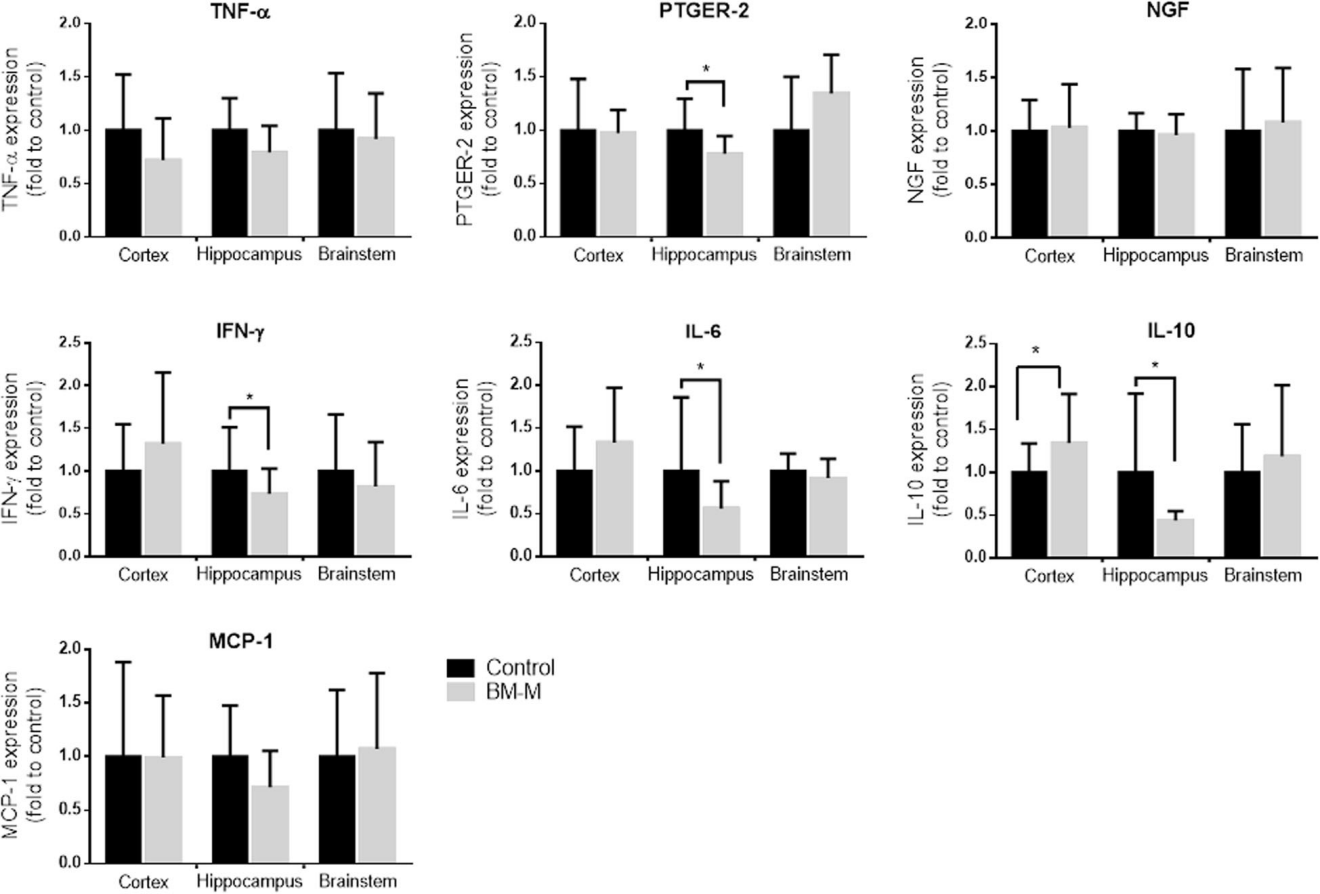
Treatment with BM-M resulted in reduced levels in inflammation markers in the hippocampus of APP/PS1 treated mice. mRNA expression of inflammation markers PTGER2, IFN-γ, IL-6 and IL-10 was reduced in the hippocampus of APP/PS1 which received BM-M (*n* = 6) compared to PBS injected control group (*n* = 6). Values were normalized to 36B4 level. Bar graphs display the mean ± SEM (error bars). Statistical significances are represented as *P < 0.05

Expression of the following genes was significantly reduced in the hippocampus of the transplanted group compared to controls: IL-6 (44%, *p* < 0.05), IFN-γ (26%, *p* < 0.05), PTGER-2 (18%, *p* < 0.012) (Fig. 7).

IL-10 expression was lowered in hippocampus (56%, *p* < 0.02) but elevated in the cortex (26%, *p* < 0.04). These results suggest that there is a decrease in neuroinflammation –particularly in the hippocampus– of the mice that received BM-M.

**Fig. 8:**
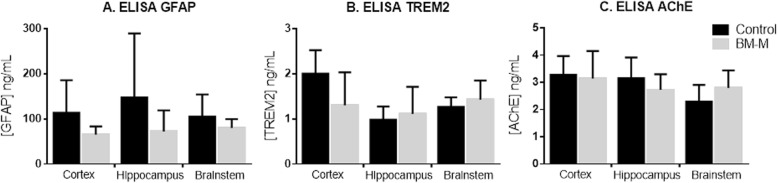
Treatment with BM-M did not change protein level of GFAP, TREM2 or acetylcholine (AchE) of APP/PS1 treated mice in any of the analysed brain regions (cortex, hippocampus and brainstem). ELISA results are displayed as means ± SEM (error bars). Statistical significances are represented as *P < 0.05

No changes were observed for: Neuronal Growth Factor (NGF) gene expression or protein levels of acetylcholine (Neurotrophic support); monocyte chemoattractant protein 1 (MCP-1) gene expression (chemotaxis); TREM-2 (phagocytotic activity) and GFAP protein levels (Fig. 8).

## Discussion

In previous studies, we demonstrated that microglia cells can be reliably generated in vitro [20]. It should be noted that while in this study, the level of CD206+ in BM-M was comparatively low compared to other ‘classic’ M2 markers, Microglia are generally found to be lower expressing for CD206 compared to blood macrophages [21]. There are three studies that have some similarity [10–13]. However, these either use an exogenous injection of Aβ as an AD animal model (which is associated with brain regional limitations) or use a different cell type (monocytes) or different age ranges. In the current study setting, we used a transgenic mouse model that shows accumulation of Aβ throughout time in several regions of the brain, moreover we have used BM-M cells and not monocytes, which have a higher resemblance with the microglia cells that are present in the brain as they are more differentiated and primed.

In a previous study, we tracked enhanced green fluorescent protein (eGFP) marked microglia intravenously transplanted to the brains of aged mice 28 days after transplantation [13]. We are therefore confident that the transplanted BM-M reach the site of investigation. In the present study, we have the advantage of obtaining results with cells that are more representative of a future clinical study, i.e., non-eGFP labelled. On the other hand this brings a limitation, since it is not possible to obtain a quantification relationship between the number of cells that reached the brain and the observed effects. What cannot be determined however, is to what extent the observed effects are directly attributable to ‘standard’ activity by the BM-M themselves or alternatively to secondary immunological reactions associated with the (potentially transient) presence of BM-M.

### Amyloid clearance

Our data shows that systemically transplanted BM-M are associated with a decrease in Aβ plaque size, especially in the hippocampus. We assume that BM-M transplantation improved the clearance of the diffuse halo of larger plaques: Aβ dense-core plaques are usually surrounded by more soluble diffuse Aβ material [22, 23] a neuropathological feature of AD consistently observable in transgenic mouse models such as APP/PS1 [17]. In contrast, Aβ pE3 is more hydrophobic, with higher aggregation propensity and stability [24] and more resistant to degradation [25–27] – all factors that may explain the lack of changes observed in the content of the Aβ pE3 peptide following a single dose BM-M transplant.

### Neuroinflammation

Neuroinflammation is one of the main hallmarks of AD [28]. Factors IL-6, IL-10, IFN-γ and PTGER-2 that are generally observed to be elevated in AD patients [29–31] were found to be reduced in the hippocampus of BM-M transplanted mice.

While expression of IL-10 was reduced in the hippocampus, it was elevated in cortex of the treated mice. While we cannot currently account for the observation, it is line with a general finding:

### Hippocampus

Many observed effects (reduction in average Aβ_[37-42]_ plaque size; increase in small IBA-1^+^cells numbers; reduction of neuroinflammation-associated gene expression) were statistically proven only the hippocampus region.

Microglia are particularly prevalent in the hippocampus [32], and regulated by their microenvironment [33]. Thus, the consistently stronger effect we tend to observe may be due to a quicker propagation of anti-inflammatory cytokines produced by the transplanted M2 BM-M.

The one departure from this trend was the reduction of reactive astrocytes following BM-M-transplants, which was significantly more pronounced in the brainstem, not in the hippocampus.

## Conclusion

Our results provide further evidence how BM-M transplantation may be impacting hallmarks of AD pathology: the observed reduction in average Aβ_[37-42]_ plaque size; and in some neuroinflammation-associated gene expression while coupled with an increase in small IBA-1^+^ cells numbers may point to a potential therapeutic utility in microglia-focussed regenerative medicine.

While we could mainly demonstrate the observed effects for the hippocampus, larger trials are needed to verify whether these effects are lasting and ubiquitous with respect to AD pathology.

## Data Availability

The datasets used and/or analysed during the current study are available from the corresponding author on reasonable request.

## Abbreviations

AChE: Acetylcholinesterase
AD: Alzheimer’s disease
Aβ: Amyloid beta
Aβ-pE3: Pyroglutamate-modified Aβ peptide
BM-M: Bone marrow derived macrophages
CNS: Central nervous system
eGFP: Green fluorescent protein (eGFP)
GFAP: Glial fibrillary acidic protein
MCP-1: Monocyte chemoattractant protein 1
NGF: Neuronal Growth Factor
PBS: Phosphate buffered saline
TREM-2: Triggering receptor expressed on myeloid cells 2
